# Heart Rate Variability to Automatically Identify Hyperbaric States Considering Respiratory Component

**DOI:** 10.3390/s24020447

**Published:** 2024-01-11

**Authors:** María Dolores Peláez-Coca, Alberto Hernando, María Teresa Lozano, Juan Bolea, David Izquierdo, Carlos Sánchez

**Affiliations:** 1 Centro Universitario de la Defensa de Zaragoza, 50090 Zaragoza, Spain; mayte.lozano@unizar.es (M.T.L.); jbolea@unizar.es (J.B.); 2 BSICoS Group, I3A Institute, University of Zaragoza, IIS Aragón, 50009 Zaragoza, Spain; alberto.hernandosanz@ioon.es (A.H.); cstapia@unizar.es (C.S.); 3 GTF Group, I3A Institute, University of Zaragoza, 50009 Zaragoza, Spain; d.izquierdo@unizar.es

**Keywords:** hyperbaric environments, autonomic nervous system, heart rate variability, subject classification, orthogonal subspace projection

## Abstract

This study’s primary objective was to identify individuals whose physiological responses deviated from the rest of the study population by automatically monitoring atmospheric pressure levels to which they are exposed and using parameters derived from their heart rate variability (HRV). To achieve this, 28 volunteers were placed in a dry hyperbaric chamber, where they experienced varying pressures from 1 to 5 atmospheres, with five sequential stops lasting five minutes each at different atmospheric pressures. The HRV was dissected into two components: the respiratory component, which is linked to respiration; and the residual component, which is influenced by factors beyond respiration. Nine parameters were assessed, including the respiratory rate, four classic HRV temporal parameters, and four frequency parameters. A k-nearest neighbors classifier based on cosine distance successfully identified the atmospheric pressures to which the subjects were exposed to. The classifier achieved an 88.5% accuracy rate in distinguishing between the 5 atm and 3 atm stages using only four features: respiratory rate, heart rate, and two frequency parameters associated with the subjects’ sympathetic responses. Furthermore, the study identified 6 out of 28 subjects as having atypical responses across all pressure levels when compared to the majority. Interestingly, two of these subjects stood out in terms of gender and having less prior diving experience, but they still exhibited normal responses to immersion. This suggests the potential for establishing distinct safety protocols for divers based on their previous experience and gender.

## 1. Introduction

The core premise of this study is centered around the notion that changes within the autonomic nervous system (ANS), which is responsible for facilitating a diver’s adjustment to dynamic hyperbaric conditions, can be measured non-invasively through the recording of physiological signals. These ANS modifications can be investigated by analyzing the variability in heart rate, which is derived from the electrocardiographic (ECG) signal [1] and is known as heart rate variability (HRV). The ANS is composed of two divisions: the sympathetic nervous system and the parasympathetic, or vagal, nervous system. An analysis of the HRV spectra unveiled two primary components: a high-frequency (HF) component attributed to respiratory sinus arrhythmia and a low-frequency (LF) component that reflects the activity of both the sympathetic and parasympathetic nervous systems. The power within the HF band is commonly utilized as a marker for parasympathetic activity. Normalized power within the LF band, along with the ratio between LF and HF power, is often employed as a measure of sympathovagal balance [2]. In addition to HRV, the respiratory signal presents another compelling avenue for investigating the ANS. This signal can be directly recorded or extracted from the ECG [3,4,5,6].

Some studies have confirmed that changes in respiratory patterns can significantly affect the spectral characteristics of HRV [7], thus influencing the interpretation of sympathetic or vagal activations [8,9]. In the conventional analysis of HRV, individuals with a respiratory rate exceeding 0.4 Hz (the upper limit of the high-frequency (HF) band) or lower than 0.15 Hz (the upper limit of the low-frequency (LF) band) are typically excluded to avoid erroneous assessments of the ANS response. To address this challenge, this study employed an orthogonal subspace projection (OSP) method [10]. By effectively separating the respiratory influences that are linearly associated with HRV, the OSP method enables a more accurate estimation of the sympathovagal balance by providing insights into the extent to which the respiratory component is reflected in HRV. This methodology has been previously validated in the examination of ANS response under induced states of worry and mindfulness [11], as well as with the implementation of pharmacological blockades to manipulate the sympathetic and parasympathetic branches [10].

The ANS response has been extensively examined in various studies via the utilization of data from hyperbaric chambers. Unlike actual dives, hyperbaric chambers enable the precise control of atmospheric pressure without the need for immersion. This controlled environment allows for the isolated investigation of pressure effects, thus eliminating the influence of other external variables. The response of the ANS has been widely analyzed in multiple works, where the conditions of high atmospheric pressures in hyperbaric chambers have been simulated without the need to actually immerse people in water [12,13,14]. Their results point to an increase in the power related to HF bands, and they are associated with an increase in the parasympathetic activity. Another conclusion of the studies in hyperbaric environments is the reduction in the HR [12,13,14], although there are also studies in which this trend was not observed [14]. The number of studies analyzing the activity of the ANS during immersion in open waters is smaller due to the implicit difficulty of obtaining adequate registers. In these immersions, in contrast with the expected increase in the sympathetic activity produced by the diving reflex and cold water stimulation [15,16], results have shown an increase in the parasympathetic activity (HF power) [17,18].

Previous research that has employed OSP methods on a hyperbaric database was documented in [19,20]. The findings revealed that parasympathetic activity gradually increases until reaching the maximum pressure of 5 atm, and this is followed by a subsequent decrease until the protocol’s conclusion at 1 atm. Similarly, sympathetic activity demonstrates a comparable pattern, displaying an initial rise from the immersion’s onset to the 5 atm stage, which is followed by a sudden decline until reaching its minimum levels during the last two stages (3 atm and 1 atm). Therefore, by utilizing HRV analysis with OSP techniques, the ANS response within hyperbaric chambers can be accurately characterized, thereby overcoming the limitations associated with respiratory rate restrictions that would have otherwise necessitated the exclusion of certain data points.

Diving is an adventurous activity that carries inherent risks such as decompression syndrome, narcosis, hyperoxia, nasal, auditory, or pulmonary barotraumas, etc. When diving with compressed air, the body’s tissues absorb nitrogen, the amount of which depends on the depth reached and the duration spent at maximum depth. To ensure safe ascent, this dissolved nitrogen must be gradually eliminated by incorporating decompression stops. These stops are meticulously planned based on decompression tables established in 1980 for recreational diving, and they are typically applicable to dives of up to 5 atm. It is important to note that these standardized tables cater to the general population without accounting for variations in sex, age, weight, diving expertise, as well as other individual factors.

Enhancing safety during dives involves adhering to important precautions, such as planning dives according to decompression tables, avoiding solo diving, and utilizing communication gesture codes among divers. Modern wearable devices like smartwatches hold potential in further augmenting immersion security. By monitoring the diver’s physiological response and identifying any inadequate reactions to the prevailing pressure, early warning systems can be established to prevent potential accidents during the dive. Various feature selection and classification methods based on pattern recognition [21] can be employed to accomplish this task successfully. In this study, feature selection is carried out using a wrapping method, which entails the selection of a subset of features based on the classifier’s accuracy.

The ultimate goal of the research framework of this study is to develop an automated system capable of accurately identifying the atmospheric pressure experienced by subjects. The correct identification of pressure signifies that the subject’s response aligns with that of the majority within the study population. Conversely, misidentification of the pressure indicates that the subject’s physiological response deviates from the norm, thereby potentially highlighting a non-normal reaction to immersion. Such instances serve as warnings and indicate a heightened risk of diving accidents. By achieving the precise identification of atmospheric pressure, this research aims to enhance safety measures and minimize potential risks during dives. To achieve this, a device equipped with a barometer should be used to label the class of the subject, and a sensor should be used to record the ECG, which determines the physiological state of the subject. If the classifier indicates that this physiological state corresponds to the class indicated by the barometer, the subject will be responding appropriately to the immersion.

To achieve the ultimate goal of this research, it is essential to identify which of the characteristics of the subjects can modify the response of their ANS—without this alteration being pathological or posing a risk to them—to variations in atmospheric pressure. This is the objective studied in this work. Identifying the characteristics that modify the ANS response during a dive will allow us to group subjects according to these characteristics, thus avoiding the erroneous identification of a risk state due to these variations.

## 2. Materials and Methods

### 2.1. Database

The generated database includes recordings of 28 subjects (25 males and 3 females) with a mean age of 28.73±6.39 years and with an annual average of dives of 30±14 for 27 of the 28 subjects. In addition, Subject 9 reported 200 dives per year. Moreover, 20 of the subjects were army divers. The database was recorded inside the hyperbaric chamber of the Hospital General de la Defensa en Zaragoza with the approval of the ethics committee *Comité de ética de la investigación con medicamentos de la inspección general de sanidad de la Defensa* (30 June 2015). The recordings of signals in the subjects were conducted in July 2015, from May to June 2016, and December 2016. In the designed protocol, five stages were studied. The protocol consisted of 5 min stops at 1 atm (pressure at sea level), at 3 atm (simulating 20 m depth), at 5 atm (simulating 40 m depth), and then coming back to 3 and 1 atm. These stages were named 1D , 3D , 5, 3A , and 1A (where the number reflects the pressure in standard atmospheres, and the letter D or A refers to descent or ascent, respectively). During the stop stages, the subjects remained relaxed and sat comfortably in silence without moving. The hyperbaric chamber was ventilated during the entire test to avoid changes in temperature and humidity. More details of this dataset can be found in [22]. The recordings were performed using the Nautilus device created by the University of Kaunas, Lithuania [23]. This device records three-lead ECG signals using a sampling frequency ( $f_{s}$ ) of 2000 Hz with a resolution of 24 bits. It also records the ambient temperature (accuracy ±0.1 °C, $f_{s}=50$ Hz) and pressure ( $f_{s}=250$ Hz, range 0–14 Bar, resolution 1.2 mBar, accuracy 50 mBar, response time 35 ms). The ECG is recorded with four electrodes placed on the chest: one near the right shoulder, another near the left shoulder, and the last two at the level of the navel, with the one near the right leg serving as the neutral ECG electrode.

The recordings of all the subjects were successful except for two subjects: (1) the Nautilus turned off unexpectedly during stages 3A and 1A in Subject 7; (2) the high-power noise signal observed in stage 5 for Subject 28 made the parameters of that stage unreliable.

The HRV parameters under investigation may be influenced by fluctuations in ambient temperature. The median and interquartile range values of the hyperbaric chamber temperature at each stage are shown in Table 1.

### 2.2. Respiratory Information from the ECG Signals

Respiratory data can be derived from the ECG signal. The initial stage involves obtaining all the derived respiration signals from the ECG (EDR). Next, an algorithm is employed to combine the information from all EDR signals for the purpose of estimating the respiratory rate.

The method utilized to estimate the respiratory rate from the ECG signal, as described in [24], capitalizes on respiration-induced changes in the ECG signal’s morphology. This is achieved through the use of three EDR signals: the upward slope of the R-wave, the downward slope of the R-wave, and the R-wave angle [24]. The method assigns to each beat occurrence the value of its two associated R-wave slope or R-wave angle.

To perform this, the first time instants associated with the maximum variation points of the ECG signal between the Q and R points ( $n_{Q_{l,i}}$ and $n_{R_{l,i}}$ ), as well as between the R and S points ( $n_{R_{l,i}}$ and $n_{S_{l,i}}$ ), were computed as follows:

(1) $$n_{U_{l,i}}=\max_{n\in [n_{Q_{l,i}},n_{R_{l,i}}]}\left\{\left|l_{l}^{\prime }\left(n\right)\right|\right\},$$

(2) $$n_{D_{l,i}}=\max_{n\in [n_{R_{l,i}},n_{S_{l,i}}]}\left\{\left|l_{l}^{\prime }\left(n\right)\right|\right\},$$

where $l^{\prime }$ is the first derivative of lead *l* and *i* indicates the beat index.

Then, a straight line was fitted to the ECG signal by least squares in two 8 ms length intervals: one centered at $n_{U_{l,i}}$ and the other at $n_{D_{l,i}}$ . The slopes of these lines—denoted as $I_{US_{l,i}}$ and $I_{DS_{l,i}}$ , respectively—were determined.

The R-wave angle was also used to derive the respiratory rate. This angle corresponds to the smallest one formed by the straight lines that define $I_{US_{l,i}}$ and $I_{DS_{l,i}}$ [25]. The equation that defines this angle is as follows:

(3) $$\phi_{R_{l,i}}=\arctan\left|\frac{I_{US_{l,i}}-I_{DS_{l,i}}}{0.4(6.25+I_{US_{l,i}}\cdot I_{DS_{l,i}})}\right|.$$

**Figure 1:**
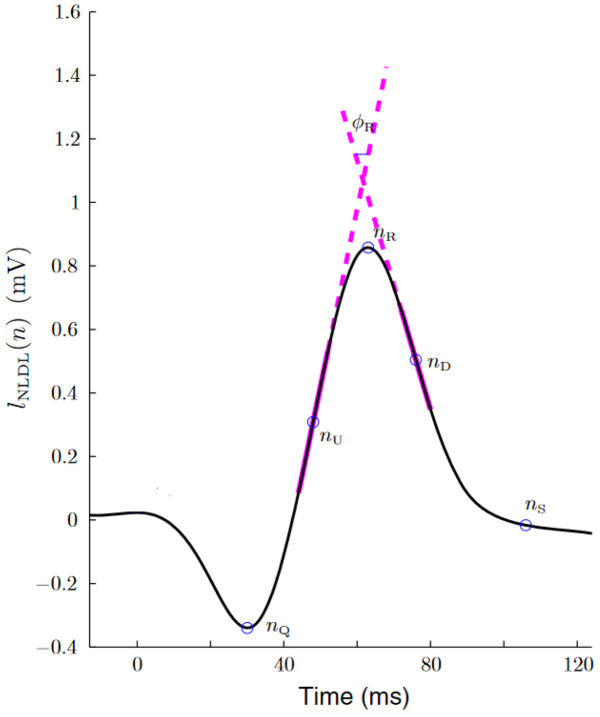
Estimation of the up slope, down slope and R-wave angle, as extracted from [24]. The thick magenta lines represent the two straight lines from which the slope series were obtained. The R-wave angle series were obtained from the smallest angle formed by these two lines.

Figure 1 shows an example of this algorithm over a QRS complex.

An EDR signal was generated for each one of the QRS slopes series by assigning to each beat occurrence ( $R_{l,i}$ ), the value of its associated QRS slope:

(4) $$d_{US,DS_{l}}^{u}\left(n\right)=\sum_{i}I_{US,DS_{l,i}}\cdot \delta \left(n-R_{l,i}\right),$$

(5) $$d_{R_{l}}^{u}\left(n\right)=\sum_{i}\phi_{R_{l,i}}\cdot \delta \left(n-R_{l,i}\right).$$

The method assigned to each beat occurrence the value of its two associated R-wave slopes or R-wave angle. These signals were unevenly sampled, so it was necessary to resample them at 4 Hz for the purpose of standardization.

**Figure 2:**
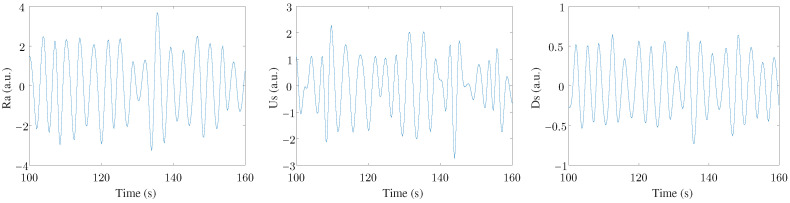
An example of the three EDR signals extracted from one ECG lead.

Finally, a MAD-based outlier rejection and a band-pass filter (with cut-off frequencies of 0.07–1 Hz) were applied to study only the frequency range where the respiratory rate was expected to be found [5]. Therefore, the three filtered EDR signals were labeled Ra, Us, and Ds. An example of these three signals can be seen in Figure 2. Three leads were registered in this study, with three EDR signals estimated for each lead. This arrangement resulted in nine final EDR signals used in the ensemble to extract respiratory information.

The fusion algorithm, as based on [24], was applied to the nine EDR signals (*j* = 1…9) to estimate the respiratory rate ( $F_{R}^{X}$ ), with X ∈ [H,P], from the peaked-conditioned averaged spectra. A power spectral density, denoted as $S_{j,k}^{X}\left(f\right)$ , was estimated every 5 s from the *k*th 40 s running window, and this was achieved using Welch’s periodogram with sub-windows of 12 s and a 50% overlap for each EDR signal (*j*). The location of the largest peak, denoted as $f_{I}^{X}\left(j,k\right)$ , was determined for each $S_{j,k}^{X}\left(f\right)$ . A reference interval $\Omega_{R}^{X}\left(j,k\right)$ was subsequently established as follows:

(6) $$\Omega_{R}^{X}\left(j,k\right)=\left[F_{R}^{X}\left(j,k-1\right)-\delta ,F_{R}^{X}\left(j,k-1\right)+2\delta \right],$$

where $F_{R}^{X}\left(k-1\right)$ is the respiratory rate estimated from the previous ( k−1 ) window and δ=0.1 .

All peaks larger than 85% of $f_{I}^{X}\left(j,k\right)$ within $\Omega_{R}^{X}\left(j,k\right)$ were detected, and $f_{II}^{X}\left(j,k\right)$ was selected as the nearest peak to $F_{R}^{X}\left(j,k-1\right)$ since respiratory variations in 5 s are supposed to be slow. Note that $f_{II}^{X}\left(j,k\right)$ could be the same as $f_{I}^{X}\left(j,k\right)$ if the largest peak was also the nearest peak to $F_{R}^{X}\left(j,k-1\right)$ .

A measure of peakness was subsequently obtained from $S_{j,k}^{X}\left(f\right)$ as the percentage of power around $f_{II}^{X}\left(j,k\right)$ with respect to the reference interval $\Omega_{R}^{X}\left(j,k\right)$ .

Then, a peaked-conditioned average spectrum, $\bar{S_{k}^{X}\left(f\right)}$ , was obtained by averaging those $S_{j,k}^{X}\left(f\right)$ that were sufficiently peaked:

(7) $$\bar{S_{k}^{X}\left(f\right)}=\sum_{l=-L_{s}}^{L_{s}}\sum_{j=1}^{N_{s}}\chi_{j,k-l}^{A}\cdot \chi_{j,k-l}^{B}\cdot S_{j,k-l}^{X}\left(f\right),$$

where $L_{s}$ was set to 2 in order to average a maximum of 5 spectra for each EDR and PDR signal, as in [24]. $N_{s}$ is the number of signals (9 for ECG and 3 for PPG), and $\chi_{j,k-l}^{A}$ and $\chi_{j,k-l}^{B}$ are the two criteria used to determine whether the power spectrum $S_{j,k-l}^{X}\left(f\right)$ was sufficiently peaked:

(8) $$\chi_{j,k}^{A}=\left\{\begin{array}{cc} 1, & P_{j,k}^{X}\geq 85\\ 0, & otherwise \end{array}\right.,$$

(9) $$\chi_{j,k}^{B}=\left\{\begin{array}{cc} 1, & P_{j,k}^{X}\geq \max_{j}\left(P_{j,k}^{X}\right)-\lambda \\ 0, & otherwise \end{array}\right..$$

Therefore, only those $S_{j,k}^{X}\left(f\right)$ whose peakness, $P_{j,k}^{X}$ , was above 85% and had a total power close to the maximum ( λ=0.05 ) were averaged.

Consequently, the respiratory rate was estimated as the maximum of $\bar{S_{k}^{X}\left(f\right)}$ as follows:

(10) $$F_{R}^{X}\left(k\right)=\arg\max_{f}\bar{S_{k}^{X}\left(f\right)}.$$

### 2.3. Time Parameters of the HRV Signal

A low-pass FIR filter was applied, with a cutoff frequency of 0.03 Hz [26], to the ECG signal to eliminate the baseline interference by subtracting it from the original signal. A wavelet-based algorithm was applied to the second frontal bipolar lead of the ECG signal to detect the heartbeat [27]. Ectopic beats, as well as missed and false detections, were identified and rectified in accordance with that described in [28]. This process resulted in the precise localization of the QRS complexes in the ECG, and the intervals between the consecutive R-waves formed the RR time series. Subsequently, the investigation applied a time-varying integral pulse frequency modulation model, the same as detailed in [29], to assess the impact of the autonomic nervous system (ANS) on the occurrence of beats. This model compensates for the influence of the mean heart rate on the modulating signal, thereby providing a more accurate representation of ANS activity. Through the application of this model, an instantaneous heart rate signal (HR) was derived at a sampling rate of 4 Hz. The mean heart rate (mHR) was obtained by low-pass filtering the HR with a cut-off frequency of 0.03 Hz. Finally, the heart rate variability (HRV) signal was obtained as the difference between the following two terms: HRV = HR − mHR.

Four time parameters were computed, from the beat to beat time series, as the mean value of the last four minutes selected for each stage (i.e., 1D , 3D , 5, 3A , and 1A ):

-
$\bar{\mathrm{NN}}$ : the median value of the normal-to-normal (NN) intervals between the fiducial points (units of time: *s*);
-
$\bar{\mathrm{IQRNN}}$ : the interquartile range of NN as a measure of statistical dispersion of all NN intervals (units of time: *s*);
-
$\bar{\mathrm{RMSSD}}$ : calculated as the square root of the mean of the squares of successive differences between adjacent NN intervals (units of time: *s*);
-
$\bar{\mathrm{pNN}50}$ : the number of pairs of successive NN intervals that differ by more than 50 ms, which are then divided by the total number of NN intervals (measurement units: %).

### 2.4. Analysis of HRV Using Orthogonal Subspace Projections

The inclusion of the respiratory signal was essential to enhance the analysis of the ANS. Specifically, it helps to capture the respiratory sinus arrhythmia, which synchronizes with respiration. Consequently, it becomes crucial to account for the relationship between respiratory rate and the parasympathetic system in our analysis. As a result, participants with a respiratory rate falling outside the range of 0.15 Hz (the upper limit of the LF band) to 0.4 Hz (the upper limit of the HF band) are typically excluded from HRV studies to prevent potential misinterpretations concerning ANS activity, as was described in [8]. However, the utilization of the OSP method in this paper obviated the need for such exclusions.

OSP is a technique designed to break down the HRV signal into two distinct components: the respiratory component, which encompasses the variations linearly associated with respiration; and the residual component, which describes the dynamics influenced by mechanisms other than respiration. The residual component embodies dynamics regulated by the sympathetic nervous system and other potential vagal modulators unrelated to respiration, as referenced in [10,11]. For this method to work effectively, both the HRV signal and the respiratory signal are required, and this is achieved by assuming that the respiratory signal induces fluctuations in the HRV signal, as was outlined in [30]. Notably, one of the EDR signals utilized for extracting the respiratory rate also serves as the respiratory signal (see Section 2.2).

To isolate all the HR dynamics linearly tied to respiration, a subspace denoted as V was constructed using the respiratory signal and its delayed versions, as was detailed in [10]. Subsequently, the HRV signal was projected onto this respiratory subspace V , thereby allowing for the description of all HRV dynamics that were linearly linked to respiration in the respiratory component, referred to as HRV_R_. Simultaneously, an orthogonal component, which represented HR modulators other than respiration, was computed as ${\mathrm{HRV}}_{⊥}$ = HRV − HRV_R_.

The relative power of each component ( $P_{R}$ for the respiratory component and $P_{⊥}$ for the residual component) indicated the extent to which respiration and the HR share information. When $P_{R}>>P_{⊥}$ , it suggests that most HR variations can be explained by changes in respiration and vice versa. Moreover, $P_{R}$ can serve as an index for assessing respiratory sinus arrhythmia (RSA), and due to the association between RSA and vagal tone, $P_{R}$ can be interpreted as a marker for parasympathetic activity.

Finally, four frequency parameters were defined as the mean value of the last four minutes that were selected for each stage ( 1D , 3D , 5, 3A , and 1A ).

-
$\bar{P_{R}}$ : the relative power of respiratory component (measurement units: arbitrary units, a.u.);
-
$\bar{P_{⊥}}$ : the relative power of the residual component (measurement units: a.u.);
-
$\bar{P_{LF_{⊥}}}$ : the power of the residual component in the LF band that could be interpreted as a marker of the sympathetic system (0.04–0.15 Hz; measurement units: a.u.);
-
$\bar{P_{HF_{⊥}}}$ : the power of the residual component in the HF band that could be interpreted as a marker of the parasympathetic system (0.15–0.4 Hz; measurement units: a.u.).

### 2.5. Statistical Analysis

In order to minimize the effects of the intersubject variability, the relative change ( R ) of each parameter ( Y ) with respect to the reference stage ( 1D ) for each studied stage was calculated as follows:

(11) $$R\left(Y_{S}\right)=\frac{Y_{S}-Y_{1D}}{Y_{S}+Y_{1D}},$$

where 1D is the reference state and *S* can be 3D , 5, 3A , or 1A .

The parameters were referenced to the basal state to minimize the effects of the intersubject variance. The Shapiro–Wilk test was used to check the normality of the ratios $R(Y_{S})$ . When the normal distribution of one ratio was verified, the t-Student paired test was applied. When not, the Wilcoxon paired test was applied. A *p*-value of < α defines the significance in value with respect to basal state 1D , where the significance level α can be 0.05 , 0.01 , or 0.001 . This test allows for identifying the significant differences in each parameter for each stage with respect to the basal state. Finally, a test using ANOVA statistics with a Bonferroni correction for multiple comparisons was applied to assess the differences between the estimated relative changes in the four stages. When the normal distribution of one relative change in the considered group was not verified, a Friedman test was applied instead of the ANOVA test. These tests were applied to the relative change in each parameter in each of the following four stages: 3D , 5, 3A and 1A .

### 2.6. Feature Selection and Classification

The objective of this study is to automatically identify the atmospheric pressure to which a subject is exposed based on changes, with respect to the basal state, in the diverse parameters extracted from the HRV.

The MATLAB R2017b Classification Learner App allows to perform multi-class classification with 27 types of classifiers and three validation methods. This app can be used to perform a first approximation and to select the classifiers that give the best response for the study population [31,32,33,34].

Among the great diversity of classifiers, in this study, we propose four of them to identify the four hyperbaric states to which the subjects were exposed. To select these four classifiers, the following procedure was implemented: A classification of stages 5 and 1A , i.e., the two stages most distant in pressure, was performed using the MATLAB Classification Learner App, thereby obtaining the accuracy of the 27 classifiers of the app. A K-fold validation was used ( k=28 , matching with the number of subjects). For the rest of the study, the four classifiers with the highest accuracy were selected: a linear discriminant analysis (LDA) classifier, a medium Gaussian support vector machine (SVM) classifier, a k-nearest neighbors (KNN) classifier based on cosine distance, and a discriminant ensemble classifier (DEC). All classifiers had a binary output that indicated whether the feature values correspond to the evaluated state or not. A true positive (TP) was considered when the classifier assigned the state that matches the pressure to which the subject was subjected. A true negative (TN) was reached when the classifier indicated that the subject did not correspond to the evaluated state and, indeed, when the subject was under a pressure different from the evaluated state. Accuracy was determined as TN+TP divided by the total cases and multiplied by 100.

A feature selector known as the wrapping method was employed. This method involves the following steps:

1. The training and validating of a classifier were performed for each of the nine features using leave-one-out validation. In the study population, one subject was selected from all of the evaluated states, and this subject constituted the test group. The remaining subjects (27 in total) formed the training group.
2. The feature with the highest accuracy was selected as the first feature ( $F=\{F_{1}\}$ , where *F* is the set of selected features). If more than one feature obtained the maximum classification, one of them was randomly selected.
3. The training and validating of classifiers using a leave-one-out approach with the following two features: the previously selected one and each of the remaining features.
4. Selecting the two features with the highest accuracy ( $F=\{F_{1},F_{2}\}$ ).
5. Repeating Steps 3 and 4 while progressively adding more features in the sequence ( $F=F_{1},F_{2},\ldots ,F_{f}$ , where f=9 represents the maximum number of features that can be selected).

This method has been applied with each of the four classifiers and for the identification of the following set of classes: 3D and 5 (C.3D-5); 5 and 3A (C.5-3A); 5 and 1A (C.5-1A); and 3A and 1A (C.3A-1A). A final set with three classes was considered, where the first class was formed by unifying states 3D and 3A into a class called C.3DA, the second class was formed by Stage 5, and the third class was formed by 1A (C.3DA-5-1A).

### 2.7. Anomaly Subject Identification Algorithm

The study population predominantly consisted of young, healthy men, with a few individuals varying in gender and possessing extensive prior diving experience. The primary objective of this work was to determine whether a classifier can identify subjects exhibiting an anomalous response compared to the majority within the population.

To address the potential limitation of a somewhat narrow study population for the stated objective, the following algorithm was implemented to identify subjects with a hyperbaric environment response that was different from the majority: Classifiers with two states are trained utilizing four different classifiers. Each of these classifiers consider a variable number of features, ranging from 1 to 9. This implies that, for each pair of the states studied, every subject has 72 results (2 × 4 × 9 = 72). To identify subjects with an anomalous response, only the results corresponding to classifiers exceeding a 70% precision are considered out of these 72 results. The subjects identified as having an anomalous response are those most frequently misclassified compared to the results obtained by the rest of the population. This algorithm ensures that the identification of the anomalous subjects is not based on the results of a single classifier but on the accumulation of classification errors (i.e., those that are misclassified).

## 3. Results

**Figure 3:**
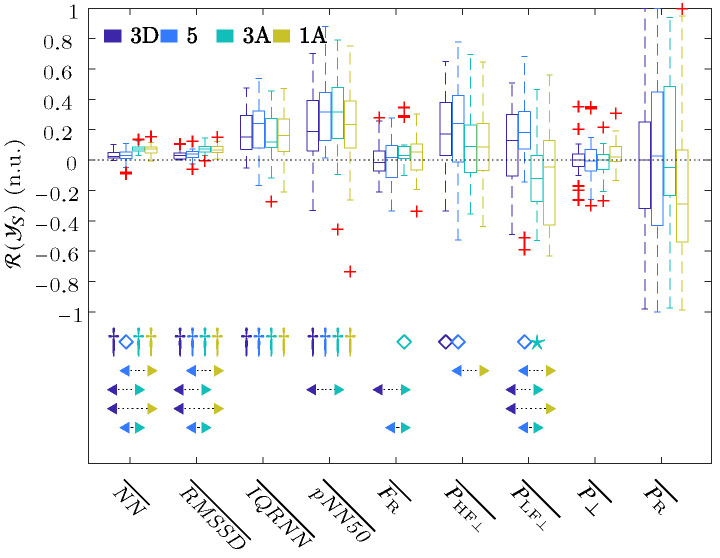
Boxplots of all the parameters of the ECG signal. The significance level α of the t-Student or Wilcoxon test was indicated with ★ for α=0.05 , ⋄ for α=0.01 , and † for α=0.001 . The arrows indicate the statistically significant differences between the compared groups, and this was achieved using ANOVA or Friedman statistics with the Bonferroni correction for multiple comparisons tests when the median value of one stage (arrow start) was significantly higher or lower than the other (arrow end). The colors of the start and end of the arrows indicate the stages analyzed.

The challenge of this study was the automatic identification of various hyperbaric stages. In order to reduce the impact of intersubject variance, all parameters were referenced with respect to a $Y_{1D}$ baseline stage using Equation (11). The boxplot in Figure 3 shows the described ratios of the respiratory rate obtained from ECG, as well as the time and frequency parameters of the HRV that were extracted using the OSP method, and this was performed for each stage referenced to the baseline 1D .

These results showed a significant increase in all the time parameters relative to the baseline, as well as among the descending ( 3D and 5) and ascending ( 3A and 1A ) stages. These results showed an increase in $F_{R}$ , which was significant for Stage 3A with respect to the baseline and also with respect to Stages 3D and 5. The $F_{R}$ , NN , and RMSSD reached their maximum values for Stage 1A , whereas IQRNN and pNN50 reached a maximum increase at Stage 5 and 3A , respectively. For the frequency domain results, $P_{HF_{⊥}}$ increased its value in all stages with respect to the basal state, but it was only significant for Stages 3A and 5. This increase reached its maximum at the deepest, i.e., Stage 5. $P_{LF_{⊥}}$ increased its value with respect to the baseline during descent, i.e., Stages 3A and 5, and then decreased in value during the ascent, i.e., Stages 3A and 1A . When comparing stages, significant changes were obtained between each of the two ascending stages with each of the two descending ones. No changes in the residual power component $P_{⊥}$ were observed, except for a small increase in Stage 1A , while there was a large dispersion in the changes in the respiratory power components, $P_{R}$ , with respect to the basal stage.

### Feature Selection and Classification

The following five sets of classes were considered for automatic identification: C.3D-5; C.5-3A; C.5-1A; C.3A-1A; and C.3DA-5-1A (Section 2.6). Due to problems in recording the ECG signal, as described in Section 2, Subject 7 only became part of the population for the set of classes C.3D-5, and Subject 28 was only part of the population for the set of classes C.3A-1A.

**Figure 4:**
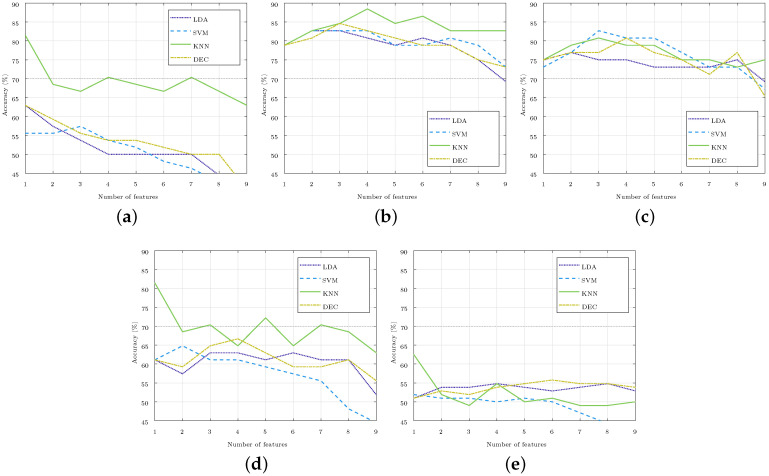
Accuracy of the subject stage identification as the number of selected features rose in each of the four classifiers used. The 70% accuracy was highlighted in the images with a gray horizontal line. (**a**) Stages 3D and 5 (C.3D-5). (**b**) Stages 5 and 3A (C.5-3A). (**c**) Stages 5 and 1A (C.5-1A). (**d**) Stages 3A and 1A (C.3A-1A). (**e**) Stages 3D , 5, 3A , and 1A (C.3DA-5-1A).

The method of feature selection was repeated for each of the four classifiers (LDA, SVM, KNN, and DEC) and for each of the five set of classes considered (C.X-Y, where X and Y stand for the stages compared). Figure 4 illustrates the accuracy obtained in the identification in each of the cases considered, which was achieved by increasing the number of the selected features, starting from one to nine. For the three-class classification, like C.3DA-5-1A, the accuracy was well below 65%, so it iswas considered that these classes could not be separated with current features, and these three-class classifiers were not considered in the rest of the paper. For the two-class classifiers, optimal results were those obtained with one, three, or four features due to both their high accuracy and low number of features.

Table 2 shows the order of the features obtained in the selection process. In this table, the feature sets with the best accuracy for each of the class sets are highlighted in blue. In the highlighted cases, the accuracy was higher than 70%, whereby the maximum of a 88.5% accuracy was reached with the two-class classifier C.5-3A with four features. This table also shows the subjects misclassified in the classifiers that obtained the best accuracy. If a subject was misclassified for the two classes considered, its number appears duplicated in the table.

**Figure 5:**
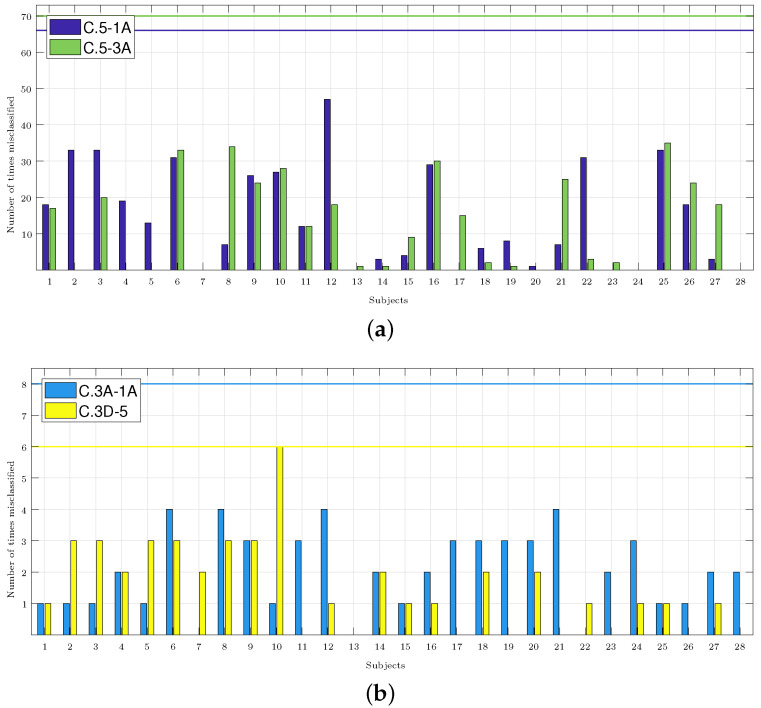
Number of times each subject was misclassified. The caption of each panel indicates the number of cases considered, i.e., those whose accuracy exceeded 70% and those multiplied by the two classes considered in each classifier. The maximum possible number of misclassifications considered in each panel is highlighted in the graphs with a horizontal line in the color associated with each set of classes. (**a**) C.5-1*A*: 66 cases. C.5-3*A*: 70 cases. (**b**) C.3*A*-1*A*: 8 cases. C.3*D*-5: 6 cases.

Figure 5 shows how many times each subject was misclassified, and it shows the accumulation of the results of all the classifiers that obtained an accuracy higher than 70% (these were most of the results in C.5-3A and C.5-1A, but just few were found in C.3A-1A and C.3D-5, as shown in Figure 4). In total, for each pair of classes, there were four different classifiers with a number of features that varied from 1 to 9, thus producing a total of 72 (2 classes × 4 classifiers × 9 features) classification results for each subject. Thus, for example, in the C.3D-5 classifiers, the accuracy only exceeded 70% for one, four, and seven features for the KNN classifier, so it was considered that the subjects may have been misclassified a maximum of 6 times (2 classes × 1 classifier × 3 features). The number of classification results considered for each subject in each pair of classes is indicated in the caption of each panel.

In this paragraph, we compare the results obtained by applying the methodology from Section 2.7, and the aim is to identify the subjects with a response different from the majority population (i.e., those who were misclassified), with the results obtained using what is considered the best classifier in each group of the analyzed classes. In Figure 5a, for the C.5-1A classifiers, Subjects 2, 3, 6, 9, 10, 12, 16, 22, and 25 were misclassified more than 20 times. Comparing this result with the one obtained by the best classifier (Table 2), it was shown that it classified Subjects 6 and 16 well, but it failed to classify Subjects 4 and 26. Only Subject 12 was misclassified in more than 40 occasions. For the C.5-3A classifiers, Subjects 3, 9, and 21 were misidentified more than 20 times, and this would then be added to the results of the best classifier. In Figure 5b for C.3A-1A, it was shown that Subjects 1, 2, 3, and 5 were misclassified once with the best classifier (Table 2). These four subjects were not army divers. In Figure 5b, if we consider the subjects that were misclassified on 3 or more occasions, the number of misclassified subjects was reduced to 7, and then to 10 for the C.3D-5 classifiers. In Section 4, the possible cause for these subjects being misclassified is analyzed.

## 4. Discussion

This study aims to automatically identify the atmospheric pressure to which a diver is exposed to. An incorrect classification of a subject may indicate that the subject is not responding adequately to the increased pressure, which would allow one to take premature actions against possible accidents during dives in hyperbaric environments. To achieve this, a database was constructed using parameters extracted from the ECG signals of 28 subjects, and these were recorded during five designated hyperbaric stages. The respiratory rate and HRV time parameters were extracted, and four HRV frequency parameters were also extracted by the OSP method.

To ensure an accurate analysis of the HRV parameters, it was crucial to eliminate the variations in ambient temperature that could potentially impact the parameters being studied. Table 1 illustrates that an increase of 4 atm in atmospheric pressure results in an approximate 3 °C rise in ambient temperature. Interestingly, within the hyperbaric chamber, there was only one instance of temperature decrease, specifically from 5 to 3A . This decrease in temperature triggered the reflex activation of sympathetic vasoconstrictor nerves, leading to cutaneous vasoconstriction and reduced blood flow to the skin [35]. However, this sympathetic activation was not observed in the $P_{LF_{⊥}}$ parameter depicted in Figure 3. Before returning to 1 atm (Stage 1A ), the divers were required to undergo decompression stops lasting between 50 and 55 min. These stops allowed the divers to gradually acclimatize to the minor temperature change in relation to the preceding stage. Notably, significant temperature variations occurred between consecutive stages, except for the transition from Stages 3A to 1A .

As a secondary objective, this study sought to automate the identification of the specific hyperbaric stages experienced by subjects using a single signal, namely the ECG signal. Reducing the number of signals required for this identification would facilitate the integration of these findings into electronic devices, thereby enhancing the safety of individuals in hyperbaric environments. Such devices are more readily accepted by the general public when they have minimal impact on the subject’s comfort. However, it is important to include the respiratory signal in this study for two key reasons: it provides valuable information regarding the subject’s adaptation to the hyperbaric environment, and it is essential for the implementation of the OSP method. One limitation of relying solely on a single signal is the omission of a dedicated respiratory signal recording. To address this limitation, an algorithm was implemented to extract the respiratory rate from the ECG signals [24]. It is worth noting that this algorithm was tested against a reference device, and its favorable results validate its use for estimating respiratory rates. The reported margin of error for the EDR methods was approximately 0.025 Hz in the worst case [24]. These algorithms provide sufficient accuracy through which to identify subjects with respiratory rates outside the [0.15 Hz, 0.4 Hz] range. In our database, there are nine subjects with respiratory rates below 0.15 Hz or above 0.4 Hz, which could potentially lead to the overestimation of power in the LF band or underestimation of power in the HF band when extracting the classical frequency parameters of HRV. However, the frequency parameters extracted in this study pertain to the residual component of HRV, thus capturing the modulated dynamics unrelated to respiration. As a result, none of these nine subjects needed to be excluded from the study, and the mean respiratory rate of all subjects was included as predictors in the classifiers. Furthermore, the OSP method necessitated the use of the respiratory signal to construct the subspace V and project the HRV signal onto this subspace. For the creation of subspace V , a respiratory signal derived from the ECG, specifically one of the EDRs described in Section 2.2, was employed.

To mitigate the impact of intersubject variability, the classification study was conducted using the proposed parameter ratios, which was achieved by focusing on the stages under study referenced with the subject’s baseline stage through Equation (11).

Figure 3 shows a significant increase in $\bar{\mathrm{NN}}$ , which corresponds to a decrease in HR, as well as a significant increase in the rest of time domain parameters from the baseline to the hyperbaric stage. Previous hyperbaric studies [12,13,14,36] have reported a reduction in HR, which could be attributed to the influence of pressure and the diving reflex [37,38]. This may be the cause as to why this parameter was the most frequently selected as the first feature (Table 2). The significant increase in the rest of the temporal parameters (especially in $\bar{\mathrm{RMSSD}}$ ), together with the decrease in the HR, seems to point out an increase in the parasympathetic activity or a decrease in the sympathetic one. This increased parasympathetic activity can also be observed in the frequency parameter $\bar{P_{HF_{⊥}}}$ , which is increased in all hyperbaric stages when compared to the basal stage and reaches its maximum change for Stage 5. Regarding the sympathetic system, an increase in the parameter $\bar{P_{LF_{⊥}}}$ for the pressure increase stages, i.e., 3D and 5, can be observed; meanwhile, a decrease in sympathetic activity in Stages 3A and 1A was observed, thereby coinciding with the largest increase in the time domain parameters $\bar{\mathrm{NN}}$ and $\bar{\mathrm{RMSSD}}$ . These three parameters also showed the same significant differences among the descending ( 3D and 5) and ascending ( 3A and 1A ) stages. Although several studies have shown that parasympathetic activation occurs to adapt to pressure increases [12,13,14,22,39], in our results, we can observe sympathetic activation in Stages 3D and 5 with the pressure increase. In a hyperbaric chamber, during increases in atmospheric pressure, respiratory effort increases. In addition, the environment is more humid and hostile, which can induce a slight stress in the subjects that justifies the increase in sympathetic activation. In Stages 3A and 1A , the reduction in atmospheric pressure, i.e., returning to baseline conditions, reduces the stress that the hyperbaric chamber can induce in the subject, and this is reflected in a decrease in the $\bar{P_{LF_{⊥}}}$ parameter in relation to the baseline.

The results of this study have direct application in the practice of diving. Bearing in mind that the maximum pressure reached is 5 atm, we are still within the limits of recreational diving. In these dives, there are no sudden changes in pressure, and divers go through each stage sequentially. That is why, in this work, sequential changes of 2 and 4 atm were compared. Comparing Stages 3D and 3A was ruled out since the subjects were exposed to the same pressure. A classifier with the three classes considered in the study was also included, but the results showed that, with the current features, these stages cannot be separated.

The MATLAB Classification Learner App allows us to perform multi-class classification with 27 types of classifiers. This app was used to perform a quick classification of all the considered multi-classes using the nine features and a K-fold validation, where K=28 . With the pair of classes C.5-3*A* and C.5-1*A*, the results were higher than 65%. For this paper, the four classifiers that presented the best results separating the classes C.5-1*A* were selected as they were the stages that experienced the highest change in pressure.

With the four classifiers selected (LDA, SVM, KNN, and DEC), we proceeded to separate the five sets of classes considered. The classifiers with the best results were those that separated the ascending stages C.5-3*A* and C.5-1*A*, as well as reached an accuracy of 88.5% and 82.7% (both of which with only four and three features), respectively. Selected features included $\bar{\mathrm{NN}}$ and $\bar{P_{LF_{⊥}}}$ (Table 2), which showed significant changes between Stage 5 and Stages 3A and 1A (Figure 3). As previously mentioned, these parameters showed us a dominance of the parasympathetic system over the sympathetic when the pressure decreases and until it recovers to the basal pressure.

To separate the classes C.5-3*A*, feature $F_{R}$ was also selected, which showed significant changes between these stages (Figure 3). This was something that did not happen with $P_{⊥}$ , which was the fourth feature selected to separate these stages (Table 2). An accuracy above 80% was obtained in the four classifiers used. The KNN classifier based on cosine distance exceeded 85% with the four features mentioned (Figure 4). The complexity of this classifier made it difficult to interpret the relationship between the selected features and the different stages studied, and this made it possible to identify the hyperbaric state in which the subject was immersed. Likewise, it was difficult to justify why a feature that did not show significant differences between the stages studied was essential for achieving this high accuracy.

Analogously, to separate the classes C.5-1*A*, feature $P_{⊥}$ was selected, which did not show significant changes between those stages (Figure 3). In this class, the maximum accuracy was achieved with a medium Gaussian SVM classifier (Figure 4). The medium Gaussian kernel used in this classifier made it difficult to interpret the results, as was the case with the KNN classifier.

Between Stages 3D and 5, and 3A and 1A , there were no significant differences for any of the nine parameters (Figure 3); however, they did reach an accuracy of 81.5% with one feature. Table 2 shows that the best results were achieved with features $\bar{\mathrm{pNN}50}$ and $\bar{\mathrm{IQRNN}}$ , but the same accuracy would be obtained if the selected feature were $\bar{\mathrm{NN}}$ . The classifiers managed to separate the stages using only this feature, while the addition of any other feature reduced the accuracy considerably. These accuracies were reached with the KNN classifier, thus leaving the accuracy below 65% for the C.3*D*-5 stages, and 67% for the C.3*A*-1*A* stages with the rest of the classifiers considered (Figure 4).

A limitation of this study is that the selected classifiers did not allow for an interpretation of the physiological changes underlying the atmospheric pressures to which the subjects were exposed. A future research line could be to separate these classes with methods that facilitate the characterization of a normal physiological response to immersion in hyperbaric environments.

Figure 5 shows that Subjects 3, 6, 9, 10, 16, and 25 were misclassified more than 20 times in the pair of C.5-1*A* and C.5-3*A* classes, and they were misclassified at least one time in the rest of the pair of classes. Subjects 3 and 6 were two of the three women of the sample population who entered a hyperbaric chamber for the first time and practiced diving recreationally. The average annual dives of the subjects that made up the sample was 30, while the annual dives of Subjects 3 and 6 were 14 and 4, respectively. Subject 16 was a man with only 15 annual dives. Subject 25 was an army diver, but he did not indicate the number of annual dives. Subjects 9 and 10 were men who were army divers and were familiar with the hyperbaric chamber, so their characteristics matched with the majority of the sample population. However, Subject 9 reported 200 dives per year—well above the population average. No subject indicated having had complications during the dive or derived from the dive, so it was considered that all had a normal physiological response to the dive. The results seemed to indicate that this response could be different depending on the sex or/and the previous experience of the subjects. It will be necessary to expand the database to confirm this hypothesis.

## 5. Conclusions

In this study, it was possible to automatically identify the atmospheric pressure to which the subjects were exposed, and an accuracy of 88.5% was reached in the differences between the stages at 5 atm and 3 atm. These results were achieved with just four features: $\bar{\mathrm{NN}}$ , $\bar{P_{⊥}}$ , $\bar{F_{R}}$ , and $\bar{P_{LF_{⊥}}}$ . The misclassified subjects, 6 of the 28, were identified as subjects with an altered physiological response compared to the rest of the subjects that made up the study population. Two of these subjects differed from the majority of the population in gender, and three of the subjects had much less previous diving experience. However, their response to immersion was considered normal. These findings suggest that it may be necessary to establish distinct safety protocols for diving based on divers’ prior experience and/or gender. Further studies with more subjects will be needed to verify this trend.

## Figures and Tables

**Table 1:** Hyperbaric chamber temperature at each stage. Results shown as median/interquartile range values.

	1*D*	3*D*	5	3*A*	1*A*
°C	31.3/3.2	33.7/2.5	34.3/2.4	31.8/2.8	32.8/2.8

**Table 2:** Selected features in each classifier. The features of the classifier with the best results for each pair of stages are marked in blue. The last column shows the misclassified subjects in the classifiers with the highest accuracy.

Classes	Classif.	Order of Features	Misclass. Subjects
C.3D-5	LDA	$\bar{P_{HF_{⊥}}}$ ; $\bar{\mathrm{pNN}50}$ ; $\bar{\mathrm{IQRNN}}$ ; $\bar{\mathrm{RMSSD}}$ ; $\bar{\mathrm{NN}}$ ; $\bar{P_{R}}$ ; $\bar{F_{R}}$ ; $\bar{P_{LF_{⊥}}}$ ; $\bar{P_{⊥}}$ ;	
	SVM	$\bar{\mathrm{IQRNN}}$ ; $\bar{\mathrm{pNN}50}$ ; $\bar{P_{LF_{⊥}}}$ ; $\bar{P_{R}}$ ; $\bar{P_{⊥}}$ ; $\bar{P_{HF_{⊥}}}$ ; $\bar{\mathrm{NN}}$ ; $\bar{F_{R}}$ ; $\bar{\mathrm{RMSSD}}$ ;	
	KNN	** $\bar{\mathrm{pNN}50}$ ; ** $\bar{\mathrm{NN}}$ ; $\bar{F_{R}}$ ; $\bar{P_{HF_{⊥}}}$ ; $\bar{\mathrm{RMSSD}}$ ; $\bar{P_{⊥}}$ ; $\bar{\mathrm{IQRNN}}$ ; $\bar{P_{LF_{⊥}}}$ ; $\bar{P_{R}}$ ;	2 3 4 5 6 7 8 9 10 10
	DEC	$\bar{P_{HF_{⊥}}}$ ; $\bar{\mathrm{pNN}50}$ ; $\bar{P_{R}}$ ; $\bar{P_{⊥}}$ ; $\bar{P_{LF_{⊥}}}$ ; $\bar{F_{R}}$ ; $\bar{\mathrm{RMSSD}}$ ; $\bar{\mathrm{IQRNN}}$ ; $\bar{\mathrm{NN}}$ ;	
C.5-3A	LDA	$\bar{\mathrm{NN}}$ ; $\bar{P_{LF_{⊥}}}$ ; $\bar{P_{R}}$ ; $\bar{\mathrm{RMSSD}}$ ; $\bar{\mathrm{IQRNN}}$ ; $\bar{P_{HF_{⊥}}}$ ; $\bar{F_{R}}$ ; $\bar{\mathrm{pNN}50}$ ; $\bar{P_{⊥}}$ ;	
	SVM	$\bar{\mathrm{NN}}$ ; $\bar{P_{⊥}}$ ; $\bar{P_{HF_{⊥}}}$ ; $\bar{F_{R}}$ ; $\bar{\mathrm{RMSSD}}$ ; $\bar{P_{LF_{⊥}}}$ ; $\bar{\mathrm{IQRNN}}$ ; $\bar{\mathrm{pNN}50}$ ; $\bar{P_{R}}$ ;	
	KNN	** $\bar{\mathrm{NN}}$ ; $\bar{P_{⊥}}$ ; $\bar{F_{R}}$ ; $\bar{P_{LF_{⊥}}}$ ; ** $\bar{\mathrm{RMSSD}}$ ; $\bar{P_{R}}$ ; $\bar{\mathrm{pNN}50}$ ; $\bar{P_{HF_{⊥}}}$ ; $\bar{\mathrm{IQRNN}}$ ;	6 8 10 16 25 26
	DEC	$\bar{\mathrm{NN}}$ ; $\bar{F_{R}}$ ; $\bar{P_{LF_{⊥}}}$ ; $\bar{P_{⊥}}$ ; $\bar{P_{HF_{⊥}}}$ ; $\bar{P_{R}}$ ; $\bar{\mathrm{IQRNN}}$ ; $\bar{\mathrm{RMSSD}}$ ; $\bar{\mathrm{pNN}50}$ ;	
C.5-1A	LDA	$\bar{\mathrm{NN}}$ ; $\bar{P_{HF_{⊥}}}$ ; $\bar{\mathrm{RMSSD}}$ ; $\bar{F_{R}}$ ; $\bar{\mathrm{pNN}50}$ ; $\bar{\mathrm{IQRNN}}$ ; $\bar{P_{LF_{⊥}}}$ ; $\bar{P_{⊥}}$ ; $\bar{P_{R}}$ ;	
	SVM	** $\bar{\mathrm{NN}}$ ; $\bar{P_{LF_{⊥}}}$ ; $\bar{P_{⊥}}$ ; ** $\bar{F_{R}}$ ; $\bar{P_{R}}$ ; $\bar{\mathrm{IQRNN}}$ ; $\bar{P_{HF_{⊥}}}$ ; $\bar{\mathrm{RMSSD}}$ ; $\bar{\mathrm{pNN}50}$ ;	2 3 4 9 10 12 22 25 26
	KNN	$\bar{\mathrm{NN}}$ ; $\bar{P_{⊥}}$ ; $\bar{\mathrm{pNN}50}$ ; $\bar{P_{LF_{⊥}}}$ ; $\bar{P_{HF_{⊥}}}$ ; $\bar{P_{R}}$ ; $\bar{\mathrm{RMSSD}}$ ; $\bar{F_{R}}$ ; $\bar{\mathrm{IQRNN}}$ ;	
	DEC	$\bar{\mathrm{NN}}$ ; $\bar{P_{⊥}}$ ; $\bar{P_{LF_{⊥}}}$ ; $\bar{\mathrm{pNN}50}$ ; $\bar{P_{HF_{⊥}}}$ ; $\bar{\mathrm{RMSSD}}$ ; $\bar{F_{R}}$ ; $\bar{\mathrm{IQRNN}}$ ; $\bar{P_{R}}$ ;	
C.3A-1A	LDA	$\bar{\mathrm{pNN}50}$ ; $\bar{P_{⊥}}$ ; $\bar{F_{R}}$ ; $\bar{\mathrm{RMSSD}}$ ; $\bar{\mathrm{NN}}$ ; $\bar{P_{HF_{⊥}}}$ ; $\bar{P_{LF_{⊥}}}$ ; $\bar{P_{R}}$ ; $\bar{\mathrm{IQRNN}}$ ;	
	SVM	$\bar{P_{R}}$ ; $\bar{P_{LF_{⊥}}}$ ; $\bar{P_{HF_{⊥}}}$ ; $\bar{P_{⊥}}$ ; $\bar{F_{R}}$ ; $\bar{\mathrm{NN}}$ ; $\bar{\mathrm{IQRNN}}$ ; $\bar{\mathrm{pNN}50}$ ; $\bar{\mathrm{RMSSD}}$ ;	
	KNN	** $\bar{\mathrm{IQRNN}}$ ; ** $\bar{\mathrm{pNN}50}$ ; $\bar{P_{LF_{⊥}}}$ ; $\bar{\mathrm{NN}}$ ; $\bar{F_{R}}$ ; $\bar{P_{⊥}}$ ; $\bar{\mathrm{RMSSD}}$ ; $\bar{P_{R}}$ ; $\bar{P_{HF_{⊥}}}$ ;	1 2 3 4 5 6 8 9 11 12
	DEC	$\bar{\mathrm{pNN}50}$ ; $\bar{P_{⊥}}$ ; $\bar{F_{R}}$ ; $\bar{\mathrm{RMSSD}}$ ; $\bar{P_{LF_{⊥}}}$ ; $\bar{P_{R}}$ ; $\bar{\mathrm{IQRNN}}$ ; $\bar{P_{HF_{⊥}}}$ ; $\bar{\mathrm{NN}}$ ;	

## Data Availability

Data are contained within the article.

## References

[1] Nitzan M., Babchenko A., Khanokh B., Landau D. (1998). The variability of the photoplethysmographic signal—A potential method for the evaluation of the autonomic nervous system. Physiol. Meas..

[2] Task Force of the European Society of Cardiology the North American Society of Pacing and Electrophysiology (1996). Heart rate variability standards of measurement, physiological interpretation, and clinical use. Circulation.

[3] Bailón R., Sörnmo L., Laguna P. (2006). A robust method for ECG-based estimation of the respiratory frequency during stress testing. IEEE Trans. Biomed. Eng..

[4] Lázaro J., Gil E., Bailón R., Mincholé A., Laguna P. (2013). Deriving respiration from photoplethysmographic pulse width. Med. Biol. Eng. Comput..

[5] Charlton P., Birrenkott D.A., Bonnici T., Pimentel M.A., Johnson A.E., Alastruey J., Tarassenko L., Watkinson P.J., Beale R., Clifton D.A. (2017). Breathing Rate Estimation from the Electrocardiogram and Photoplethysmogram: A Review. IEEE Rev. Biomed. Eng..

[6] Hayano J., Yuda E. (2019). Pitfalls of assessment of autonomic function by heart rate variability. J. Physiol. Anthropol..

[7] Zhang P.Z., Tapp W.N., Reisman S.S., Natelson B.H. (1997). Respiration response curve analysis of heart rate variability. IEEE Trans. Biomed. Eng..

[8] Hernando A., Lazaro J., Gil E., Arza Valdes A., Garzon-Rey J., Lopez-Anton R., de la Camara C., Laguna P., Aguilo J., Bailón R. (2016). Inclusion of respiratory frequency information in heart rate variability analysis for stress assessment. IEEE J. Biomed. Health Inform..

[9] Long X., Fonseca P., Haakma R., Aarts R.M., Foussier J. (2014). Spectral boundary adaptation on heart rate variability for sleep and wake classification. Int. J. Artif. Intell. Tools.

[10] Varon C., Lazaro J., Bolea J., Hernando A., Aguilo J., Gil E., Van Huffel S., Bailon R. (2019). Unconstrained Estimation of HRV Indices after Removing Respiratory Influences from Heart Rate. IEEE J. Biomed. Health Inform..

[11] Widjaja D., Caicedo A., Vlemincx E., Van Diest I., Van Huffel S. (2014). Separation of respiratory influences from the tachogram: A methodological evaluation. PLoS ONE.

[12] Barbosa E., García-Manso J.M., Martín-González J.M., Sarmiento S., Calderón F.J., Da Silva-Grigoletto M.E. (2010). Effect of Hyperbaric Pressure During Scuba Diving on Autonomic Modulation of the Cardiac Response: Application of the Continuous Wavelet Transform to the Analysis of Heart Rate Variability. Mil. Med..

[13] Lund V., Laine J., Laitio T., Kentala E., Jalonen J., Scheinin H. (2003). Instantaneous beat-to-beat variability reflects vagal tone during hyperbaric hyperoxia. Undersea Hyperb. Med..

[14] Lund V., Kentala E., Scheinin H., Klossner J., Sariola-Heinonen K., Jalonen J. (2000). Hyperbaric oxygen increases parasympathetic activity in professional divers. Acta Physiol. Scand..

[15] Haddad H.A., Laursen P.B., Chollet D., Lemaître F., Ahmaidi S., Buchheit M. (2010). Effect of cold or thermoneutral water immersion on post-exercise heart rate recovery and heart rate variability indices. Auton. Neurosci..

[16] Perini R., Veicsteinas A. (2003). Heart rate variability and autonomic activity at rest and during exercise in various physiological conditions. Eur. J. Appl. Physiol..

[17] Chouchou F., Pichot V., Garet M., Barthélémy J.-C., Roche F. (2009). Dominance in cardiac parasympathetic activity during real recreational SCUBA diving. Eur. J. Appl. Physiol..

[18] Schipke J., Pelzer M. (2001). Effect of immersion, submersion, and scuba diving on heart rate variability. Br. J. Sport. Med..

[19] Hernando A., Posada-Quintero H., Peláez-Coca M.D., Gil E., Chon K.H. (2022). Autonomic Nervous System characterization in hyperbaric environments considering respiratory component and non-linear analysis of Heart Rate Variability. Comput. Methods Programs Biomed..

[20] Sánchez C., Hernando A., Bolea J., Izquierdo D., Rodríguez G., Olea A., Lozano M.T., Peláez-Coca M.D. (2023). Enhancing Safety in Hyperbaric Environments through Analysis of Autonomic Nervous System Responses: A Comparison of Dry and Humid Conditions. Sensors.

[21] Bishop C.M. (2006). Pattern Recognition and Machine Learning.

[22] Hernando A., Peláez M., Lozano M.T., Aiger M., Izquierdo D., Sanchez A., Lopez-Jurado M.I., Moura J.I., Fidalgo J., Lázaro J. (2018). Autonomic Nervous System Measurement in Hyperbaric Environments using ECG and PPG signals. IEEE J. Biomed. Health Inform..

[23] Sokas D., Gailius M., Marozas V. Diver physiology monitor and its graphical user interface. Proceedings of the International Scientific—Practical Conference, Virtual Instruments in Biomedicine.

[24] Lázaro J., Alcaine A., Romero D., Gil E., Laguna P., Pueyo E., Bailón R. (2014). Electrocardiogram derived respiratory rate from QRS slopes and R-wave angle. Ann. Biomed. Eng..

[25] Romero D., Ringborn M., Laguna P., Pueyo E. (2013). Detection and quantification of acute myocardial ischemia by morphologic evaluation of QRS changes by an angle-based method. J. Electrocardiol..

[26] Sörnmo L., Laguna P. (2005). Bioelectrical Signal Processing in Cardiac and Neurological Applications.

[27] Martinez J.P., Almeida R., Olmos S., Rocha A., Laguna P. (2004). A wavelet-based ECG delineator: Evaluation on standard databases. IEEE Trans. Biomed. Eng..

[28] Mateo J., Laguna P. (2003). Analysis of Heart Rate Variability in the Presence of Ectopic Beats Using the Heart Timing Signal. IEEE Trans. Biomed. Eng..

[29] Bailón R., Laouini G., Grao C., Orini M., Laguna P., Meste O. (2011). The Integral Pulse Frequency Modulation Model with Time-Varying Threshold: Application to Heart Rate Variability Analysis During Exercise Stress Testing. Biomed. Eng. IEEE Trans..

[30] Berntson G.G., Cacioppo J.T., Quigley K.S. (1993). Respiratory sinus arrhythmia: Autonomic origins, physiological mechanisms, and psychophysiological implications. Psychophysiology.

[31] Matlab M. Classification Learner App. https://www.mathworks.com/help/stats/classification-learner-app.html.

[32] Forsyth D. (2019). Applied Machine Learning.

[33] Han S., Li M., Ren Q. (2019). Discriminating among tectonic settings of spinel based on multiple machine learning algorithms Discriminating among tectonic settings of spinel based on multiple machine learning algorithms. Big Earth Data.

[34] Gupta S., Buriro A., Crispo B. (2019). DriverAuth: A risk-based multi-modal biometric-based driver authentication scheme for ride-sharing platforms. Comput. Secur..

[35] DeGroot D.W., Kenney W.L. (2007). Impaired defense of core temperature in aged humans during mild cold stress. Am. J. Physiol. -Regul. Integr. Comp. Physiol..

[36] Pendergast D.R., Moon R.E., Krasney J.J., Held H.E., Zamparo P. (2015). Human Physiology in an Aquatic Environment. Comprehensive Physiology.

[37] Gooden B.A. (1994). Mechanism of the human diving response. Integr. Physiol. Behav. Sci..

[38] Berry N.T., Wideman L., Rhea C.K., Labban J.D., Chon K.H., Shykoff B.E., Haran F.J., Florian J.P. (2017). Effects of prolonged and repeated immersions on heart rate variability and complexity in military divers. Undersea Hyperb. Med..

[39] Pelaez-Coca M.D., Hernando A., Lozano M.T., Sanchez C., Izquierdo D., Gil E. (2021). Photoplethysmographic Waveform and Pulse Rate Variability Analysis in Hyperbaric Environments. IEEE J. Biomed. Health Inform..

